# Discrimination of mild cognitive impairment based on involuntary changes caused in voice elements

**DOI:** 10.3389/fneur.2023.1197840

**Published:** 2023-06-21

**Authors:** Masakazu Higuchi, Mitsuteru Nakamura, Yasuhiro Omiya, Shinichi Tokuno

**Affiliations:** ^1^Department of Bioengineering, Graduate School of Engineering, The University of Tokyo, Tokyo, Japan; ^2^Graduate School of Health Innovation, Kanagawa University of Human Services, Yokosuka, Japan

**Keywords:** voice, prosody, calculational task, logistic regression analysis, mild cognitive impairment discrimination

## Abstract

In this study, the technique associated with the capturing involuntary changes in voice elements caused by diseases is applied to diagnose them and a voice index is proposed to discriminate mild cognitive impairments. The participants in this study included 399 elderly people aged 65 years or older living in Matsumoto City, Nagano Prefecture, Japan. The participants were categorized into healthy and mild cognitive impairment groups based on clinical evaluation. It was hypothesized that as dementia progressed, task performance would become more challenging, and the effects on vocal cords and prosody would change significantly. In the study, voice samples of the participants were recorded while they were engaged in mental calculational tasks and during the reading of the results of the calculations written on paper. The change in prosody during the calculation from that during reading was expressed based on the difference in the acoustics. Principal component analysis was used to aggregate groups of voice features with similar characteristics of feature differences into several principal components. These principal components were combined with logistic regression analysis to propose a voice index to discriminate different mild cognitive impairment types. Discrimination accuracies of 90% and 65% were obtained for discriminations using the proposed index on the training and verification data (obtained from a population different from the training data), respectively. Therefore, it is suggested that the proposed index may be utilized as a means for discriminating mild cognitive impairments.

## 1. Introduction

The number of people suffering from dementia continues to increase worldwide. According to the World Alzheimer Report 2015 (1) reported by Alzheimer's Disease International (ADI), the number of dementia patients worldwide in 2015 was estimated to be 46.8 million. In addition, ADI conducted a meta-analysis using dementia epidemiology surveys in countries around the world from 1980 to 2004 (2). Based on these surveys, the population of dementia patients is expected to reach 80 million by 2040.

In Japan, the number of dementia patients is rapidly increasing as the country transitions into a super-aging society. When mild cognitive impairments (MCIs) are also accounted, which can be considered as a predementia stage, one in five elderly people aged 65 years and older is expected to develop dementia by 2025 (3). The cost of managing dementia includes medical expenses and nursing and informal care that will affect the economy (4). To address this challenge, the early detection and preventive treatment of the disease have become increasingly essential. It is difficult to distinguish dementia from other diseases, such as depression in its early stages, and a judgment can be made based on multiple tests. Therefore, a simple initial screening method is required to diagnose dementia. Common cognitive ability tests in clinical practice include the mini-mental state examination (MMSE) (5), Montreal cognitive assessment (MoCA) (6), and Mini-Cog (7). However, these tests are not easy to perform, and physicians have to be involved. In addition, diagnostic imaging methods, such as computed tomography (CT) (8), magnetic resonance imaging (MRI) (9), and positron emission tomography (PET) (10) can be used, but these have drawbacks, such as high cost and invasiveness.

We have been studying techniques to diagnose depression from voice (11–13). Analysis using voice samples offers the advantages of noninvasiveness and easy and remote performances without the need for special, dedicated equipment. It has been hypothesized that dementia patients also have different vocal prosodies when compared with healthy subjects; based on this hypothesis, we have attempted to estimate cognitive impairments from voice samples (14). In this study, the MMSE score was estimated using multiple regression analysis from the voice features of patients diagnosed with dementia without considering the type of dementia. The results indicated that the MMSE score could be estimated accurately for severely demented patients. However, sufficient accuracy was not obtained for the MCI patient and healthy volunteer groups. In addition, the data used for the analysis does not contain a sufficient number of samples, and the accuracy of the unknown data was unknown. Therefore, the accuracy of estimation for the MCI group and the verification of accuracy for the unknown data are limitations of this method. MCI is an early stage of dementia, and its detection leads to early detection of dementia and is of high significance. Therefore, the purpose of this study is to propose a voice index that can accurately discriminate MCI from involuntary changes in voice features.

The field of voice analysis has become increasingly popular during the last decade owing to the rapid increase in research studies that have used it to detect neurodegenerative diseases. Martínez-Nicolás et al. conducted a systematic review of the research studies pursued over the past decade to detect Alzheimer's disease (AD) and MCI using voice features (15). They identified 35 studies whose quality could be guaranteed considering their selection criteria; most of the diagnostic studies had a diagnostic accuracy of ≥80% for MCI. They excluded studies in languages other than English and Spanish; however, it can be inferred that the concept of MCI detection based on involuntary changes in voice features is still in its infancy.

Toth et al. (16) imposed three types of oral tasks on healthy and MCI subjects, calculated the features related to the number of phonemes and voice/silence intervals from the voice samples recorded in each task, and discriminated between the two groups of subjects using several classical machine-learning methods. The accuracy of discrimination for the training data was in the range of 60%–70%.

König et al. (17) imposed four types of oral tasks on healthy subjects and MCI and AD patients, calculated task-dependent voice features from the voice samples recorded in each task, and used a support vector machine (SVM) to create classifiers from these voice features to discriminate between the two groups. The feature data were divided into training and verification data based on random sampling, and the accuracy of discrimination of the verification data was evaluated based on the classifier obtained from the training data. Therefore, MCI was discriminated with a sensitivity of approximately 80%.

Shimoda et al. (18) calculated voice features related to the prosody and silence intervals from the voice samples of healthy subjects and AD patients recorded over the telephone (including MCI to mild/moderate AD patients), and classical machine learning and statistical methods were used to discriminate between the two groups. The performance of the obtained classifier was evaluated based on the verification data, which were different from the training data. The training and verification data were obtained based on random sampling of the original voice dataset. Multiple telephonic voice samples were recorded per subject, and an accuracy of approximately 90% was obtained for each method in voice-based evaluations. In addition, subject-based evaluations yielded accuracies which were approximately equal to 100% for each method.

In other studies, multiple types of tasks were imposed on the subjects, and the burden on the subjects was heavy (16, 17). In addition, the voice features representing changes in the prosody were not used in the classifier training (16, 17). The training and verification data were obtained from the same population, and the classification performance for data from unknown populations was undetermined (17, 18). Features related to the voice and silence lengths were used, and the number of subjects was insufficient (16–18).

In recent years, an increasing number of studies investigated the possibility that dual-tasking (that is, doing two things simultaneously) contributes to MCI. Several studies have investigated whether cognitive decline influences walking during the performance of specific tasks. Additionally, Bahureksa et al. (19) conducted a systematic review of studies that illustrated an association between cognitive function and walking. This study suggested that it becomes more difficult to perform certain tasks as cognitive function declines. We correlated this phenomenon with voice features and hypothesized that as dementia progresses, task performance would have a greater impact on vocal cords and considerably change prosody.

Considering these perspectives, in our study, we recorded the voice samples of 399 elderly people, including healthy and MCI subjects, while they performed calculational tasks and read out the calculated results. We then calculated the voice features related to changes in prosody from these voice samples and proposed a discrimination index to discriminate between the two groups using statistical methods. The evaluation of the accuracy of the proposed discrimination index was conducted on a dataset obtained from a population that was different from the training data.

## 2. Method

### 2.1. Ethical considerations

This study was conducted with the approval of the research ethics committee of the University of Tokyo and Matsumoto Health Lab (University of Tokyo No. KE21-20).

### 2.2. Subjects

The subjects chosen for the study were elderly people aged 65 years or older recruited as members of the Matsumoto Health Lab, which is an organization that supports health promotion and was established with the support of Matsumoto City, Nagano Prefecture, Japan. The organization invited members to participate in the study, held an information session for interested members, and asked for their consent for participation. Members who agreed to participate in the study underwent clinical evaluations and voice recordings at a designated venue on a subsequent date.

The research data were collected once each year from 2020 to 2022. Different participants were recruited each year. For the convenience of the participants, multiple locations were set up each year for the data collection. Each participant went to a venue that was easily accessible to them and cooperated in the collection of the data. In 2020 and 2022, the data were collected at the same three venues. In 2021, research data were collected at three venues in two different locations.

### 2.3. Clinical evaluations

The subjects were clinically evaluated based on the MMSE. Each subject was classified into one of the three categories: healthy, MCI, and cognitive disease (CD), based on their MMSE scores. The cutoffs of the MMSE scores were the values commonly used in clinical practice. That is, a subject with an MMSE score of ≥28 was judged to be healthy, a subject with scores of ≥24 and ≤27 was judged to be an MCI patient, and a subject with a score of ≤23 was judged to be a CD patient. In this study, subjects classified as CD patients were also treated as MCI patients.

### 2.4. Voice recordings

At each venue, the voice samples were recorded in rooms with relatively good soundproofing. Subjects were assigned a calculation task in which they continued to subtract 7 from 100 up to 65 in their heads, and they were asked to vocalize the answer, which was then recorded. Even if they made a mistake in the calculation, the subjects were asked to continue the calculation until they uttered five answers, regardless of whether they were correct or not. Some subjects gave up on the calculation partway through or had to start over; therefore, the number of utterances was sometimes less than or more than five. The reason for choosing this calculation task was that the MMSE also involves the same task. In addition to the calculation of voice samples, voice samples during the reading of the numerical sequence 93, 86, 79, 72, and 65 (the results of the calculation that subtracts 7 from 100 up to 65) were also recorded; the subjects were asked to read the sequence aloud from a printed paper. In each case, the voice recording was preceded by the reading of the numerical sequence. The numerical sequence was read aloud twice. The calculation voice and reading voice samples of the numerical sequence were recorded in separate rooms. A portable recorder (R-26, Roland, Japan) and pin microphone (ME52W, Olympus, Japan) were used for the recordings. The recording environment was 24-bit at 96 kHz.

### 2.5. Voice analysis

The voice samples were volume-normalized to account for differences in volume in the recording environment. As the calculation voice samples often included utterances, such as “uhm,” during the vocalization of the answer, only the utterance part of the answer was extracted from the calculation voice samples to exclude these noise types from the analysis. Therefore, the calculation voice samples were divided based on the intervals between the utterances of the responses. The reading voice samples were used in their entirety without being divided. Thereafter, the voice features related to the prosody were calculated from each voice sample recording. We used openSMILE (20) to calculate the voice features. openSMILE is a platform that comprehensively calculates voice features. The platform provides a set of scripts that automatically calculates the set of various features from speech. The following set of scripts was used in this study:

- Large openSMILE emotion feature set
- INTERSPEECH 2009 Emotion Challenge feature set
- INTERSPEECH 2010 Paralinguistic Challenge feature set
- INTERSPEECH 2011 Speaker State Challenge feature set
- INTERSPEECH 2012 Speaker Trait Challenge feature set
- INTERSPEECH 2013 ComParE Challenge feature set

This set was used to calculate the low-order features (fast Fourier transform (FFT), Mel-frequency cepstral coefficient (MFCC), voiced sound probability, zero-crossing rate, energy, fundamental frequency F0, and others) from a voice recording. The low-order features were calculated for each frame; thus, a time series was obtained. Thereafter, the moving average of the time series of the low-order features was calculated, followed by the first (difference) and second derivatives. Finally, high-order features (mean, maximum, minimum, centroid, quartile, variance, kurtosis, skewness, and others) were calculated as statistical quantities from each of the three series processed in time.

Each calculation voice sample was divided into several utterance intervals; therefore, the features were obtained for each utterance interval and averaged. There were two reading voice samples for each subject; therefore, the features for each were obtained and averaged. We then considered the reading of the numerical sequence as a base. Based on the extent to which the vocalization changed with respect to the base while performing the mental arithmetic calculations, we calculated the difference in value (amount of change from the base; henceforth, referred to as the “feature difference”) between the numerical sequence and calculated voice features. Thereafter, we used the data obtained from the subjects in 2020 as the training data to apply a learning algorithm based on statistical techniques to the calculated feature differences.

When the number of explanatory variables is too large, overlearning is expected to occur. Therefore, we selected the features that were effective for discrimination in advance. First, the features in which there were significant differences between the calculation and reading voice states were selected using a generalized linear mixed model (21). This model used data that were repeatedly measured under multiple conditions and analyzed the differences caused by the conditions considering random effects between the subjects. To maximize the benefits from the iterative data, in this step, the features obtained from the calculation and reading voice samples were not averaged for each subject; instead, the features for each utterance interval of calculation voice samples and for each reading voice sample were used in the analysis.

Next, we selected features whose differences correlated with the MMSE score.

Finally, the discriminative performance of the feature difference between the healthy and MCI groups for each feature was calculated based on the area under the receiver operating characteristic (ROC) curve (AUC), and the features for the number of subjects from the training data were selected in the descending AUC order. Some of the selected features were essentially similar; therefore, principal component analysis was applied to aggregate features with similar characteristics of feature differences into several principal components. The features were selected based on the number of subjects when features were selected based on AUC because if the number of features is greater than the number of subjects, the covariance matrix of the feature difference will drop in rank, and principal component analysis will not be applied. Thereafter, regularized logistic regression analysis (22) was applied using the obtained principal components as explanatory variables.

In this study, regularization was conducted using the L1 norm (lasso estimation). The optimal value of the regularization parameter was determined through cross-validation of the training data. During cross-validation, the data were divided randomly; therefore, the regularization parameter will have a different value each time the algorithm is executed. Therefore, the learning result will also be different each time the algorithm is executed. Therefore, to stabilize the learning result, the learning algorithm was executed multiple times, and the results obtained in each trial were averaged to obtain the final learning result. The learning result was the regression coefficient of the principal component and the logistic function value with the linear sum of the principal component and the regression coefficient as the variable was used as the MCI discrimination index (MCIDI). The discrimination threshold used to separate the healthy and MCI groups was set to the value that minimized the balanced error rate (BER), which is the harmonic mean of the false positive and negative rates (23). The evaluation of the accuracy of the MCIDI was conducted based on the sensitivity, specificity, positive predictive value, negative predictive value, F-measure, and accuracy of the training data, AUC, and cross-validation. We also evaluated the versatility of the MCIDI using the data obtained from the subjects in 2021 and 2022 as the verification data.

Statistical analysis was conducted with the free-source software R (version 4.1.0, an official part of the Free Software Foundation's GNU project) (24).

## 3. Results

### 3.1. Subjects

The number of members who were recruited from the Matsumoto Health Lab and consented to participate in the study as subjects was 198 (150 healthy, 47 MCI, 1 CD) in 2020, 92 (76 healthy, 15 MCI, 1 CD) in 2021, and 109 (90 healthy, 18 MCI, 1 CD) in 2022 among 399 participants. Table 1 present the subject details.

**Table 1:** Details of subjects in from 2020 to 2022.

**Variable**	**Venue A Mean (Standard deviation (SD))**	**Venue B Mean (SD)**	**Venue C Mean (SD)**
Data in 2020			
Subjects, *n*	75	72	51
Age	74.07 (5.88)	72.92 (5.08)	70.96 (4.12)
Sex (male), *n*%	24 (32%)	31 (43%)	14 (27%)
Grip (kg)	29.39 (8.47)	32.46 (9.42)	30.76 (8.37)
Healthy group, *n*	59	55	36
Sex (male), *n*%	17 (29%)	27 (49%)	9 (25%)
MMSE score	29.69 (0.56)	29.58 (0.66)	29.67 (0.59)
MCI group, *n*	15	17	15
Sex (male), *n*%	6 (40%)	4 (24%)	5 (33%)
MMSE score	26.13 (0.83)	25.71 (0.77)	26.20 (0.86)
CD group, *n*	1	0	0
Sex (male), *n*%	1 (100%)	-	-
MMSE score	20 (0)	-	-
Data in 2021			
Subjects, *n*	55	15	22
Age	74.11 (5.66)	73.80 (5.83)	72.68 (4.24)
Sex (male), *n*%	25 (45%)	6 (40%)	9 (41%)
Grip (kg)	29.61 (8.83)	28.09 (9.01)	30.93 (7.78)
Height (cm)	159.74 (8.16)	156.65 (9.14)	160.02 (9.40)
Weight (kg)	59.14 (10.96)	57.68 (9.95)	60.32 (9.90)
Systolic blood pressure (mmHg)	146.65 (18.57)	142.4 (19.09)	143.95 (12.23)
Diastolic blood pressure (mmHg)	80.45 (9.91)	69.33 (9.61)	80.41 (11.25)
Healthy group, *n*	43	12	21
Sex (male), *n*%	18 (42%)	5 (42%)	9 (43%)
MMSE score	28.65 (1.93)	29.83 (0.39)	29.76 (0.44)
MCI group, *n*	11	3	1
Sex (male), *n*%	6 (55%)	1 (33%)	0 (0%)
MMSE score	25.64 (1.12)	25.67 (1.53)	26 (0)
CD group, *n*	1	0	0
Sex (male), *n*%	1 (100%)	-	-
MMSE score	23 (0)	-	-
Data in 2022			
Subjects, *n*	49	41	19
Age	73.31 (5.69)	73.32 (6.07)	69.74 (3.03)
Sex (male), *n*%	19 (39%)	21 (51%)	9 (47%)
Grip (kg)	28.53 (8.75)	31.85 (10.03)	33.45 (9.90)
Height (cm)	157.37 (9.16)	159.51 (8.43)	159.97 (8.41)
Weight (kg)	56.09 (10.68)	61.03 (10.38)	59.21 (9.47)
Systolic blood pressure (mmHg)	141.55 (16.52)	147.71 (17.07)	144.68 (13.27)
Diastolic blood pressure (mmHg)	84.51 (9.85)	83.73 (7.85)	82.58 (6.89)
Healthy group, *n*	42	31	17
Sex (male), *n*%	18 (43%)	14 (45%)	7 (41%)
MMSE score	29.40 (0.73)	29.58 (0.62)	29.88 (0.49)
MCI group, *n*	6	10	2
Sex (male), *n*%	1 (17%)	7 (70%)	2 (100%)
MMSE score	25.83 (1.17)	25.90 (0.74)	25.50 (0.71)
CD group, *n*	1	0	0
Sex (male), *n*%	0 (0%)	-	-
MMSE score	23 (0)	-	-

The mean (standard deviation) of the number of utterances of the calculation voice samples was 5.39 (1.21) in 2020, 5.09 (0.48) in 2021, and 5.23 (0.69) in 2022. The number of voice samples pertaining to the reading of the numerical sequence was 2 × 198 + 1 = 397 (only one person repeated it three times) in 2020, 2 × 92 = 184 in 2021, and 2 × 109 = 218 in 2022.

In this study, data obtained from participants in 2020 were used as training data, and data obtained from participants in 2021 and 2022 were used as verification data.

### 3.2. Feature selection

First, 15440 types of high-order features per voice sample (excluding duplicates) were obtained using the openSMILE script.

A generalized linear mixed model was applied to the 2020 data. The following models were set for each feature:


(1) $$\begin{array}{c} f_{ik}^{p}=\left(\beta_{0}^{p}+b_{i}^{p}\right)+\beta_{1}^{p}s_{ik}^{p}+\epsilon_{ik}^{p} \end{array}$$

where *f* is the feature, *s* is the binary value of either the calculation or the reading state, *p* = 1, 2, …, 15440 denotes the feature number, *i* = 1, 2, …, 198 is the number of the subject, and *k* is the utterance order of subject *i*. In addition, β_0_, β_1_ denote the fixed effects, *b*_*i*_ denotes a random effect, which represents the effect of the subject, and ϵ denotes the error. In this model, 12432 types of features were obtained when the significant features were selected at the β_1_ level of 1%.

Subsequently, we selected features with an absolute correlation coefficient value (between the feature difference and the MMSE score) greater than 0.15 and obtained 1006 types of features. Pearson's product-moment correlation coefficient was used as the correlation coefficient.

Finally, 198 types of features were selected based on the AUC. The AUC of the selected features had minimum and maximum values of 0.63 and 0.70, respectively.

### 3.3. Regularized logistic regression analysis

Principal component analysis was applied to the 198 selected features of the 2020 data, and principal components up to a cumulative contribution rate of 80% and 26 principal components were finally obtained.

**Figure 1:**
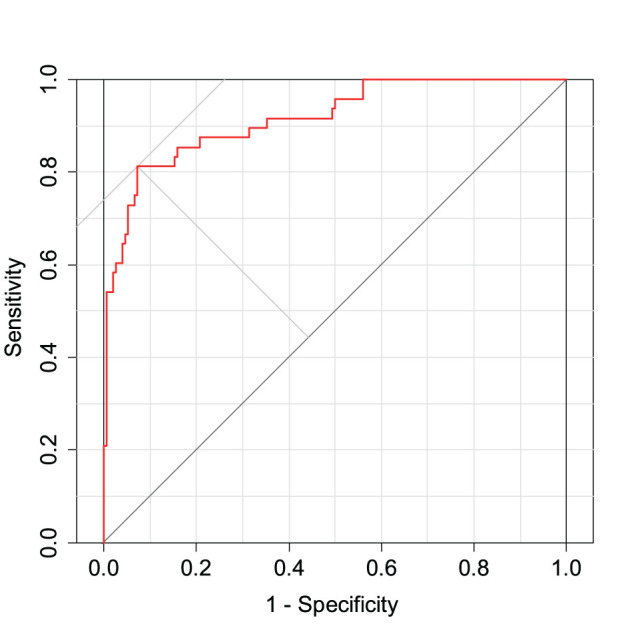
Receiver operating characteristic (ROC) curve for the mild cognitive impairment discrimination index (MCIDI) values obtained from the 2020 data (training data).

Regularized logistic regression analysis with the L1 norm was conducted 20 times on the 26 principal components, and the average value of the regression coefficients was obtained. When its mean regression coefficient was set as α_*i*_, the MCIDI was expressed in terms of the following equation,


(2) $$\begin{array}{c} MCIDI=\frac{1}{1+\exp\left(-\alpha_{0}-\sum_{i=1}^{26}\alpha_{i}PC_{i}\right)} \end{array}$$

where α_0_ is the intercept and *PC*_*i*_ is the value of the *i*th principal component. The MCIDI threshold for discriminating between the healthy and MCI groups is a value that minimizes the BER of the MCIDI values obtained for each subject in 2020 using (2); the MCIDI threshold value was approximately equal to 0.36. Table 2 lists the confusion matrix when the 2020 data were classified into the healthy and MCI groups using this threshold. The sensitivity, specificity, positive predictive value, negative predictive value, F-measure, and accuracy for the 2020 data were 0.81, 0.93, 0.78, 0.94, 0.80, and 0.90, respectively. Figure 1 depicts the ROC curve of the discrimination performance of MCIDI for the 2020 data. The AUC was 0.92.

**Table 2:** Confusion matrix when the 2020 data (training data) were discriminated based on the mild cognitive impairment discrimination index (MCIDI) threshold.

**2020 data** **(training data)**		**Predicted group**	
		**MCI group**	**Healthy group**
Actual group	MCI group	39	9
	Healthy group	11	139

**Figure 2:**
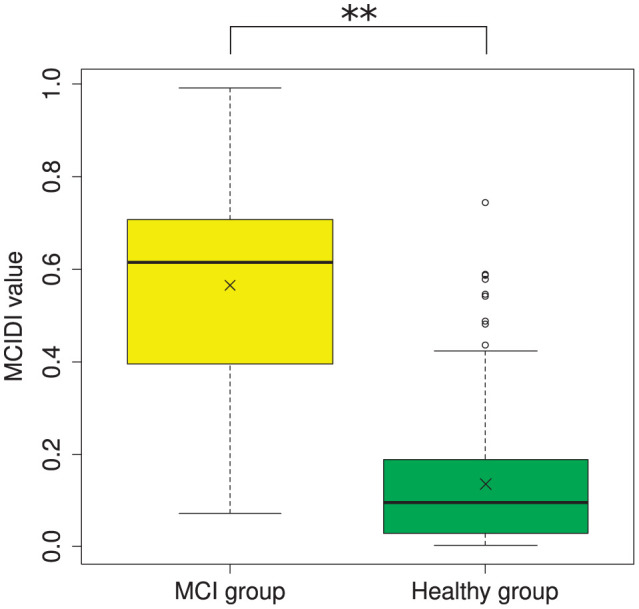
Distribution of the MCIDI values obtained from the 2020 data (training data). **p < 0.01.

Figure 2 depicts the distribution of the MCIDI values obtained for the 2020 data. A significant difference was found between the mean MCIDI values of the healthy and MCI groups in a *t*-test wherein equality of variances was not assumed (Welch's *t*-test) [*t*(56.61) = 11.03, *p* < 0.01].

Table 3 presents the *k*-fold cross-validation results for the 2020 data. The sensitivity value was higher than 0.6 and lower than 0.7. The specificity was approximately greater than 0.9. The accuracy was approximately greater than 0.8.

**Table 3:** *k*-fold cross-validation for 2020 data (training data) (*k* = 5, 10, 20, 50, 198).

***k***	**Sensitivity**	**Specificity**	**Accuracy**
5	0.60	0.87	0.81
10	0.63	0.85	0.79
20	0.63	0.85	0.79
50	0.67	0.86	0.81
198 (leave-one-out)	0.67	0.85	0.81

### 3.4. Correlation between MCIDI and MMSE scores and gender differences in MCIDI

**Figure 3:**
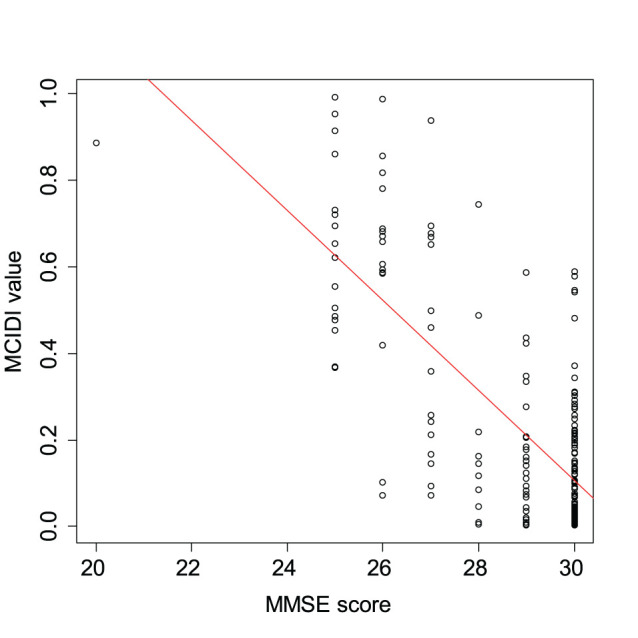
Scatterplot of the MCIDI values and mini-mental state examination (MMSE) scores obtained from the 2020 data (training data).

Figure 3 presents a scatterplot of the MCIDI values and MMSE scores obtained from the 2020 data. The red line in the figure represents the regression line for the MCIDI values. The correlation coefficient between the MCIDI value and MMSE score was −0.73 (*p* < 0.01).

**Figure 4:**
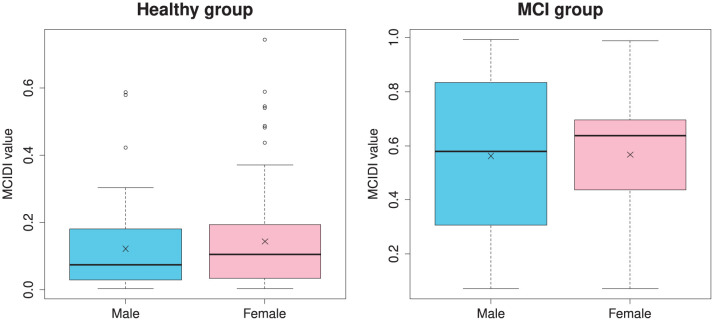
Gender-specific distribution of the MCIDI values for the healthy and MCI groups obtained from the 2020 data (training data).

Figure 4 depicts the distribution of the MCIDI values by gender for the healthy and MCI groups in the 2020 data. A *t*-test that does not assume equal variances (Welch's *t*-test) was used to compare the mean values of the MCIDI values obtained for males and females and no significant difference was observed in any group (healthy group: *t*(117.48) = −0.94, *p* = 0.35; MCI group: *t*(24.85) = −0.058, *p* = 0.95).

### 3.5. Verification

Table 4 presents the confusion matrices when the 2021 and 2022 data were used as the verification data, which were classified into the healthy and MCI groups using the MCIDI and its threshold. The sensitivity, specificity, positive predictive value, negative predictive value, F-measure, and accuracy at this time were 0.63, 0.62, 0.26, 0.89, 0.36, and 0.62 for the 2021 data and 0.53, 0.70, 0.27, 0.88, 0.36, and 0.67 for the 2022 data, respectively.

**Table 4:** Confusion matrices when the 2021 and 2022 data (verification data) were discriminated using the MCIDI threshold.

**2021 data**		**Predicted group**	
**(verification data)**		**MCI group**	**Healthy group**
Actual group	MCI group	10	6
	Healthy group	29	47
**2022 data**		**Predicted group**	
**(verification data)**		**MCI group**	**Healthy group**
Actual group	MCI group	10	9
	Healthy group	27	63

The correlation coefficient between the MCIDI value and MMSE score was −0.16 (*p* = 0.13) for the 2021 data and −0.091 (*p* = 0.35) for the 2022 data.

## 4. Discussion

Numerous studies in recent years have attempted to discriminate MCI using voice, as attempted by the present study. Most of these studies also extracted voice features from voice samples and proposed discrimination indices using various learning algorithms; their accuracy is comparable (16) to or better (17, 18) than that of the index proposed in this study. However, the accuracy evaluations of the discrimination indices used data that were obtained from the same population as the training data for verification (in other words, the training and verification data were obtained by randomly splitting the original data (17, 18), and higher accuracy accuracies were a somewhat expected outcome). It can be inferred that the design of the present study in which verification was conducted on data that were obtained from a truly different population than that for the training data, is more challenging than those of other similar studies. Other similar studies have used features related to the silence and voice lengths included in the human voice (16–18) and combined clinical assessments that were unrelated to voice and sociodemographic information, health profiles, personal and family medical histories, and others, in conjunction with voice features, to achieve high accuracy (25). However, in the present study, we only used features that were related to changes in vocal prosody. Furthermore, although other similar studies have used voice features as-is, this study used the difference from the prosody of the paired no-task voice as the change in the prosody of voice during task execution, which can be considered as a new point of focus that has not yet been explored in other similar studies. In addition, while other similar studies imposed a large number of speech tasks on subjects, which can be burdensome for them (16, 17), only two types of voice samples were recorded in this study, and the entire process lasted less than ~1 min; in this way, the burden on the subjects was small compared with that in other studies. In this study, logistic regression was used as the learning method. The reason for this was that the description of the algorithm is clearer than those of machine-learning methods. Many other similar studies used machine learning methods, such as SVM (26), random forest (27), and Light Gradient Boosting Machine (LightGBM) (28) for learning. Empirically, if the number of datasets is only a few hundred, there are no clear differences between the methods. In fact, when the analysis was performed with SVM and LightGBM instead of logistic regression, as expected, there was no clear difference in the results and the accuracy was lower than that of logistic regression.

Although we did not set clear criteria for the recruited subjects, almost all of the subjects who participated in this study were able to go to the data collection venue by themselves and were able to live their daily lives without any problems. When we actually interacted with them for data acquisition, almost all of them were able to communicate without any problems; thus, we inferred that there was almost no suspicion of other neurological diseases or Parkinson's disease. For this reason, it was considered more appropriate to treat subjects with CD based on the MMSE as MCI rather than CD.

Research on dementia detection using voice involves features that are related to voice length and silence intervals included in the human voice as a mainstream method. However, in this study, we did not use these features at all. The reason for this was to eliminate the possibility of erroneously recognizing these features as contributors toward CD because people who are not good at mental calculations will naturally have longer vocalization times. In addition, clinical evaluations based on MMSE were conducted before the voice recordings, and the numerical sequence was read out prior to the voice recording. Therefore, we were concerned that the subjects may have remembered the answers to the previous calculational task in the MMSE or perceived that the numerical sequence read earlier was a sequence of the correct results of the calculation task. However, it seemed that the subjects who were not good at calculating could not perform the calculations smoothly even for the calculation tasks conducted during the voice recordings. In fact, there was a significant weak positive correlation between the calculation task score in the MMSE and the accuracy of the calculation task during the voice recording. In addition, the subjects moved rooms after reading the numerical sequence; therefore, it was assumed that few subjects were aware that the numerical sequence contained the correct results of the calculation task.

In the selection of voice features, which is the prelearning stage for deriving the discrimination indices, approximately 80% of the features were selected using generalized linear mixed models, and it was assumed that the prosody of voice changed significantly between the calculation and readout states. Among these features, more than 8% were related to cognitive function. Therefore, the correlation between task execution and cognitive function may have been weak. In fact, the discrimination performance of the 198 types of features finally selected was approximately 0.6–0.7 in terms of the AUC. Therefore, it cannot be said that the discrimination performance of the features alone was particularly high.

Regularized logistic regression analysis was applied to the 26 principal components that were aggregated through principal component analysis to obtain an index (MCIDI) that could discriminate between the healthy and MCI groups in the training data with an AUC >0.9. In addition, the sensitivity, specificity, and accuracy were all ≥0.8; that is, the degree of accuracy was the same as those for the indices proposed in other similar studies. There was a relatively high correlation between the MCIDI and MMSE scores of the training data. The target variable used for learning was binary; therefore, the target variable did not directly include the MMSE score information. However, the feature difference that was correlated to the MMSE score was used as an explanatory variable. Therefore, it is inferred that an index that correlates with the MMSE score was obtained. In addition, there was no gender difference in the MCIDI values of both the healthy and MCI groups in the training data. However, males in the MCI group yielded a wider distribution of MCIDI values than that yielded by females.

The individual discrimination performances of the 198 types of feature differences, which are the basis of the principal components, were not particularly good. However, the accuracy of the learning result was good. Therefore, it was necessary to consider the possibility of overlearning. We cross-validated the training data to investigate this possibility, and the results indicated that the specificity exceeded 0.8 for any number of divisions, but the sensitivity was only approximately 0.6, and there was a possibility of overlearning. However, in the training data of the present study, the number of subjects in the MCI group was approximately 30% of the number of subjects in the healthy group, and it was thought that it was expected that the specificity would be high. In a comparison with the verification data obtained from a population different from the training data, consideration of the cross-validation results indicated that the calculated sensitivity was a reasonable result. The specificity for the 2021 data was low compared with the cross-validation results, but relatively high for the 2022 data as well as the cross-validation results. The accuracy was not good and was in the range of 0.6–0.7. However, in general, the accuracy of verification for data that were obtained from a population that was different from the training data tended to be lower than that for the training data. We infer that an accuracy rate >0.6 as part of a challenging research design with few other examples was a reasonable result. Although the populations used for the training and verification data were different, they belonged to the same city; therefore, we expected a certain degree of verification accuracy. However, the results we obtained were somewhat unexpected. One possible reason is that due to the coronavirus 2019 pandemic, the number of subjects was not sufficient for verification and the data collection period was divided into two; this made it impossible to align the measurement venues and resulted in differences in the recording environment.

A limitation of the present study is that subject conditions were insufficient. Approximately 30% of Alzheimer's patients are known to develop MCI between the ages of 40 and 65. Conversely, in Japan, the prevalence of MCI in the elderly aged 65 years and over is 13%, but it decreases rapidly at lower ages. Therefore, a large population is required to collect a sufficient number of prevalent young people. Hence, we targeted mental disorders in the elderly in this study. However, it would be interesting to investigate whether our proposed MCIDI would be effective for subjects younger than 65 years. If the MCIDI is effective in younger age groups, it may be effective for the early detection of Alzheimer's disease in younger people.

Secondly, the MMSE sensitivity was insufficient. Therefore, potential MCI subjects were classified as healthy subjects, and it was inferred that the learning did not effectively capture the changes in the vocal cords that were unique to MCI. In fact, the output of the logistic function that was obtained through learning strictly represented the probability that a subject had MCI, and it is appropriate to set the discrimination threshold to separate the healthy group from the MCI group at 0.5. However, in the present study, the discrimination threshold was set to the value that minimized the BER. That is, most of the MCI group members were determined to be healthy group members. In this sense, the learning result of the present study indicated a fit that was biased toward healthy individuals. To address this problem, the quality of MCI subjects will have to be improved in combination with the MoCA.

Thirdly, we discussed the relationship between voice features and MCI. Several voice features that were selected during learning were associated with the Mel frequency (29) and MFCC (30). The feature set of openSMILE used in the present study included several features calculated on the Mel-frequency axis. Thus, it is inferred that several features that were related to the Mel frequency were inevitably selected. The selected features were obtained in a broad band from low to high on the Mel-frequency axis, and it was difficult to specify which frequency band particularly responded to MCI. Therefore, a future task is to identify voice features that match the physiological changes caused by MCI. Conversely, we also studied the detection of Alzheimer's disease and dementia with Lewy bodies using voice cited in (31, 32). One of our future studies is to investigate the extent to which the features responding to Alzheimer's disease and dementia with Lewy bodies obtained in these studies differ from the features responding to MCI in this study.

Fourthly, we discussed the selection of appropriate speech tasks for assignment to the subjects. This study employed a computational task, but the pauses in the voice could not be used to avoid MCI misclassifications for those who were not good at mental calculations. Ideally, people who are not good at mental calculations should be excluded, but it is difficult to distinguish whether they are really good or not at mental calculations or are unable to perform calculations due to MCI. In this sense, a word-recall task may have been more appropriate as a task.

Finally, we discussed the system design that anticipates future developments in this field. In this study, we did not consider any errors in the results of the mental arithmetic calculations. Our objective was to understand the differences in voice features between utterances that used the brain's functions for calculation and those that did not; therefore, the content of the utterances themselves was not an issue. However, if the contents of the utterances were different from those of the paired utterances, then the feature differences would increase; thus, there is a possibility that this may have a large effect. One way to avoid this possibility is to control the content of the utterances using techniques such as voice recognition. However, these advanced techniques take time to process. In the future, a smartphone app can be used to process the results of this study as a MCI discrimination system. However, real-time voice recognition with current smartphone specifications is expensive; it is thus inferred that a system design that includes voice recognition cannot be easily proposed at present.

## 5. Conclusion

In this study, we proposed a voice index to facilitate the discrimination of MCIs based on involuntary changes in voice. The participants of this research were elderly individuals aged 65 years or older. They were divided into healthy and MCI groups based on clinical evaluations. For the study, we acquired the participants' voice samples during a mental calculation task and during the reading of the correct answers to the calculation tasks written on paper, and we expressed the prosody changes in the calculated voice samples compared with the reading of voice samples based on acoustic feature differences. We used principal component analysis to aggregate groups of features with similar characteristics of feature differences into several principal components. Thereafter, we combined the principal components with logistic regression analysis to propose a voice index to discriminate MCI. The proposed index discriminated the training data into healthy and MCI groups with an accuracy of 90% and the verification data that were obtained from a population other than the training data into healthy and MCI groups with an accuracy of 65%. In a challenging study design with few other examples, an accuracy >60% is a reasonable result; however, in general, this is not a sufficient accuracy outcome. The reason for this is that the consideration of a variety of factors, such as subject inclusion criteria, task assignments, features selected (or not selected), and confounding factors, may not be appropriate, and there is still room for additional considerations. However, it is suggested that the proposed index may be utilized as a new index for discriminating MCI.

## Data availability statement

The raw data supporting the conclusions of this article will be made available by the authors, without undue reservation.

## Ethics statement

The studies involving human participants were reviewed and approved by the Research Ethics Committee of the University of Tokyo and Matsumoto Health Lab. The patients/participants provided their written informed consent to participate in this study.

## Author contributions

ST was responsible for the design of the clinical study. MH analyzed the data and wrote the manuscript. MN and YO facilitated the interpretation of the study findings. All authors participated in the editing, revision of the final version of the manuscript, have read, and agreed to the published version of the manuscript. All authors contributed to the article and approved the submitted version.
